# Supplementary material for: A Global Screen for Assembly State Changes of the Mitotic Proteome by SEC-SWATH-MS
Source: Cell Syst. 2020 Feb 26;10(2):133–155.e6. doi: 10.1016/j.cels.2020.01.001 (PMC7042714; doi:10.1016/j.cels.2020.01.001)

Q9P032 | NDUF4_HUMAN | NDUFAF4 C6orf66 HRPAP20 HSPC125 My013
Monomer MW [kDa]: 20.266 Monomer expected elution fraction: 54

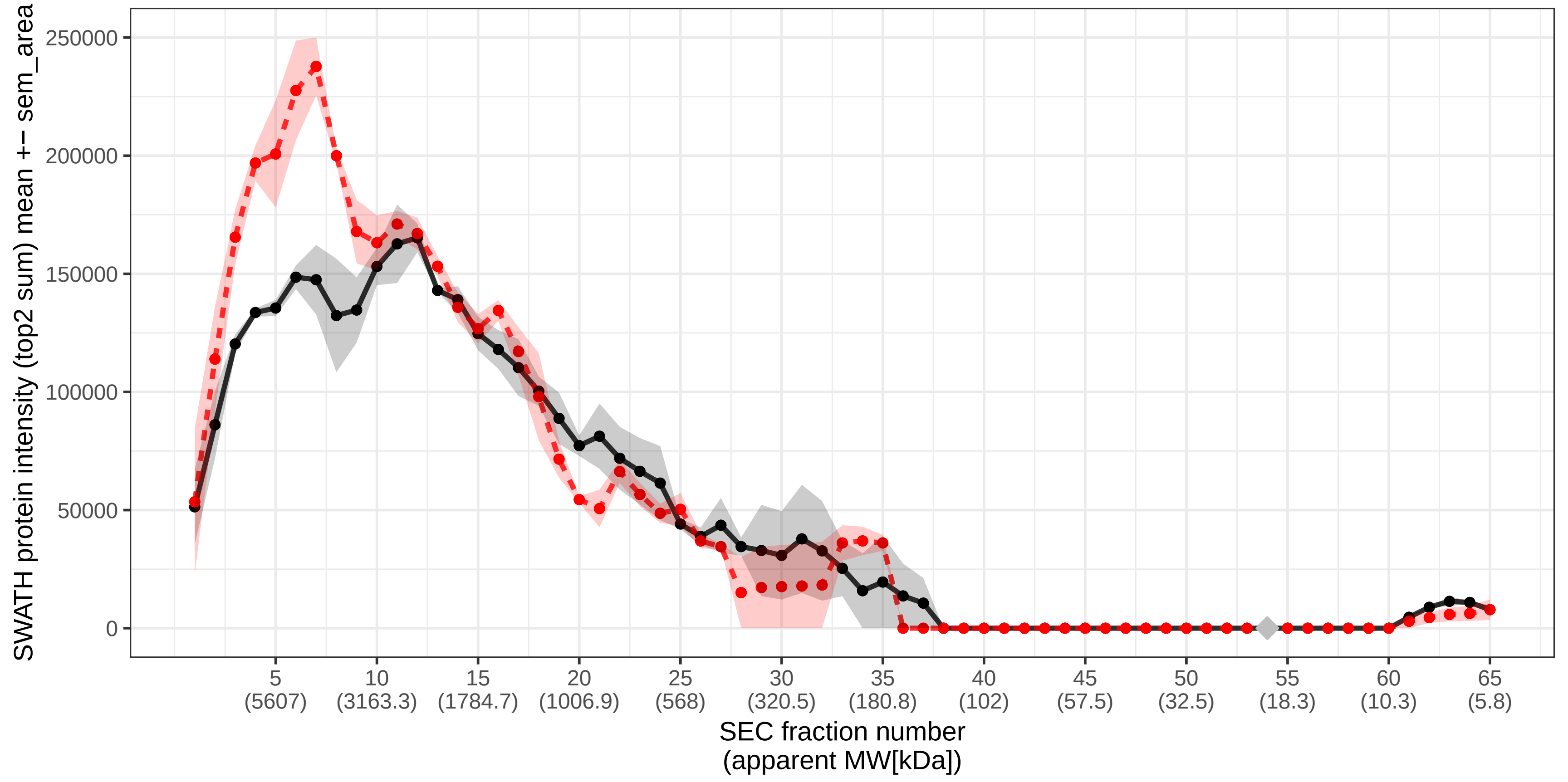

Supplement: Data S1. SEC-SWATH-MS Protein Chromatograms, Related to Figure 1 [file mmc6.zip › SECchrom_Q9P032_NDUF4_HUMAN_NDUFAF4_C6orf66_HRPAP20_HSPC125.pdf]
